# The Grievances of Poor-Law Medical Officers

**Published:** 1915-10-30

**Authors:** 


### THE GRIEVANCES OF POOR-LAW MEDICAL OFFICERS.

The Poor-Law Medical Officers' Association has prepared for presentation to the Departmental Committee of the Local Government Board engaged upon the examination of the Poor-Law Orders a long memorandum setting forth the points the Association considers require amendment. The memorandum is strengthened by an appendix giving examples of the way in which the present Orders render it difficult for the Poor-Law medical officer to carry out his duties efficiently.

Many of the Poor-Law Orders affecting medical officers were drawn up sixty or seventy years ago. It is, therefore, not surprising that many of them no longer meet present-day requirements. Under the Medical Appointments Order of May, 1857, the district medical officer is required to be resident within his district. This is a very real hardship in many cases. The increase in the means of intercommunication has been so great that this restriction is no longer necessary, and the Association urges, with good reason, that the Order should be varied so as to read "resident within his district or within short distance therefrom." Another Order which has long become obsolete is that dealing with extra fees payable to the district medical officer for certain emergency operations. The Order was issued in July, 1847, and the only operations mentioned therein are amputations and herniotomy for strangulated hernias. The Association points out that the field of operative work has been largely increased, and that Poor-Law medical officers are now expected to perform many other operations, some as matters of urgency and others as part of their ordinary work. The Association has drawn up a list of these operations with an appropriate fee for each. The list includes laparotomy for acute abdominal conditions, trephining, the ligature of main arteries for hæmorrhage, tracheotomy, lumbar puncture, the removal of adenoids, and others. The Association also suggests that a fee of £1 should be paid to the anæsthetist and to any medical man required to assist in any of the major operations, and that the scale should apply to non-resident workhouse medical officers as well as to district medical officers.

### Inadequate Remuneration.

Other long-standing grievances which the memorandum suggests the Local Government Board should redress are inadequacy of remuneration, the provision of drugs by medical officers, and the ever-increasing demands for certificates of all kinds. The Association draws attention to cases where the Local Government Board has protested against the inadequacy of the remuneration offered by certain Boards of Guardians to their medical officers, and complains that these protests have not been followed by any effective action. It considers that the Board should obtain powers to enforce its recommendations in such cases, and should lay down a scale of minimum salaries with a provision for automatic increases at periodic intervals when the work has been satisfactorily performed. It is also strongly of opinion that the Board should refuse its assent to any contract requiring the medical officer to supply drugs and dressings at his own expense.

### The Doctor and the Master.

Another grievance to which we have often drawn attention, and one which militates greatly against the efficient performance of his work, is the subordinate position the workhouse medical officer holds as regards the workhouse master in the administration of the sick wards. The Association holds that the medical officer should be supreme in the management of these wards. This is a view that no unprejudiced person with a knowledge of the condition of things in mixed workhouses is likely to contest. It is the most important of all the reforms advocated, and the one which is most urgently required in the interests of the sick inmates.

Several other points of less importance generally are touched upon. It is suggested that when a district medical officer is required to attend a patient who is subsequently found not to be a pauper, and from whom expenses are recovered, the medical officer should share in the charges so recovered. The system in force at Wigan, under which a police constable can obtain an order from a relieving officer for a district medical officer to attend a person taken ill in the streets, is condemned as unfair to the Poor-Law medical officer and to the police surgeon, and as liable to lead to unnecessary delay in obtaining medical assistance. The unfairness of requiring a district medical officer, in whose district an isolation hospital has been erected, to attend its pauper inmates drawn from all parts of the Union, or to carry out special treatment of children found defective by the school medical officer, is pointed out. It is a special grievance that in some instances district medical officers have been required to carry out work, such as the estimation of errors of refraction, which cannot be properly performed by a medical man who has not devoted special study to it.

The Association is to be congratulated on the careful manner in which the memorandum has been prepared by its energetic secretary, Dr. Major Greenwood. The moderation of its demands and the practical character of the reforms suggested should ensure its success.

